# Supplementary material for: Spectroscopic disentanglement of the quantum states of highly excited Cu2
Source: Nat Commun. 2019 Jul 22;10:3270. doi: 10.1038/s41467-019-11156-2 (PMC6646321; doi:10.1038/s41467-019-11156-2)
Supplement: Supplementary file 1 — Supplementary Information [file 41467_2019_11156_MOESM1_ESM.pdf]

SPECTROSCOPIC DISENTANGLEMENT OF THE QUANTUM STATES OF HIGHLY EXCITED CU_2

Beck *et al.*

$^{63}Cu_2$			
Level	T_v	B_v	$D_v \times 10^8$
$J0_u^+$ (v =0)	37448.8417(70)	0.1156040(149)	6.49(52)
$J0_u^+$ (v =1)	37736.7189(437)	0.1147195(381)	7.222(962)
$J0_u^+$ (v =2)	38022.4414(243)	0.1138145(459)	9.45(203)
$G_{62}0_u^+$	37513.6802(84)	0.058225(12)	
$G_{63}0_u^+$	37615.60^a	0.0580^a	
$G_{64}0_u^+$	37717.4033(545)	0.05788(10)	
$G_{65}0_u^+$	37818.6986(74)	0.0574771(85)	
$G_{66}0_u^+$	37919.64^a	0.0572^a	
$G_{67}0_u^+$	38020.048(30)	0.05698(12)	
$G_{68}0_u^+$	38120.011(164)	0.0567581(962)	
$L1$	37473.0678(668)	0.1024332(428)	
$M1$	38032.871(135)	0.099883(153)	
N1	38035.144(46)	0.07834(13)	

$^{65}Cu^{63}Cu$			
Level	T_v	B_v	$D_v \times 10^8$
$J0_u^+$ (v =0)	37448.7096(95)	0.1138453(198)	7.425(836)
$J0_u^+$ (v =1)	37734.3030(79)	0.1129210(174)	4.952(677)
$J0_u^+$ (v =2)	38017.7366(81)	0.1120524(336)	12.0(25)
$G_{62}0_u^+$	37464.8515(255)	0.057551(22)	
$G_{63}0_u^+$	37567.349(467)	0.056780(228)	
$G_{64}0_u^+$	37667.66^a	0.0570^a	
$G_{65}0_u^+$	37768.355(17)	0.056719(17)	
$G_{66}0_u^+$	37868.530(168)	0.0565208(720)	
$G_{67}0_u^+$	37968.93^a	0.0562^a	
$G_{68}0_u^+$	38067.939(33)	0.056130(44)	
$L1$	37463.8282(451)	0.1009676(354)	
$O1$	38020.244(18)	0.100307(34)	

$^{63}Cu_2$ Perturbation	Strength
$< J0_u^+(v=0)\|\|G_{62}0_u^+ >$	1.153(11)
$< J0_u^+(v=0)\|\|G_{63}0_u^+ >$	1.1^b
$< J0_u^+(v=1)\|\|G_{64}0_u^+ >$	2.512(218)
$< J0_u^+(v=1)\|\|G_{65}0_u^+ >$	2.7194(71)
$< J0_u^+(v=1)\|\|G_{66}0_u^+ >$	2.6^b
$< J0_u^+(v=2)\|\|G_{67}0_u^+ >$	5.171(16)
$< J0_u^+(v=2)\|\|G_{68}0_u^+ >$	4.8045(73)
$< J0_u^+(v=0)\|\|L1 >$	1.2949(89^c
$< J0_u^+(v=2)\|\|M1 >$	0.345(11)^c
$< J0_u^+(v=2)\|\|N1 >$	0.8866(83)^c

$^{65}Cu^{63}Cu$ Perturbation	Strength
$< J0_u^+(v=0)\|\|G_{62}0_u^+ >$	1.265(14)
$< J0_u^+(v=0)\|\|G_{63}0_u^+ >$	1.061(13)
$< J0_u^+(v=1)\|\|G_{64}0_u^+ >$	2.5^b
$< J0_u^+(v=1)\|\|G_{65}0_u^+ >$	2.7288(74)
$< J0_u^+(v=1)\|\|G_{66}0_u^+ >$	2.590(13)
$< J0_u^+(v=2)\|\|G_{67}0_u^+ >$	5.2^b
$< J0_u^+(v=2)\|\|G_{68}0_u^+ >$	4.8194(66)
$< J0_u^+(v=0)\|\|L1 >$	1.1064(61)^c
$< J0_u^+(v=2)\|\|O1 >$	0.1945(88)^c
$< G_{62}0_u^+\|\|L1 >$	1.872(53)^c

Supplementary Table 1: Molecular Constants of the Deperturbed Vibronic States of $^{63}Cu_2$ and $^{65}Cu^{63}Cu$. All values in units of cm^{-1} (with one standard deviation in units of the last figure appended in parentheses). T_v: origin (sum of electronic term Te and vibrational term $G(v)$). B_v: mean value of the rotational constant B in the vibrational state v. D_v: mean value of the centrifugal distortion constant D in the vibrational state v. ^{a}interpolated values. ^{b}presumed values based on the other isotopologue. ^{c}For comparison with the determined homogeneous $G0_u^+$ $J0_u^+$ perturbation, the same perturbation model is applied for the deperturbation of the $L1, M1, N1$ and $O1$ perturber states. A definitive assignment to the perturbation class, however, requires more detailed investigations.

Constant	This work	Previous work[1]
T_e	37437.107(95)	37437.20(33)
ω_e	289.93(16)	289.40(88)
$\omega_e x_e$	1.075(53)	0.64(44)[2]
B_e	0.116062(12)	0.116218(54)
$\alpha_e \times 10^3$	0.9123(77)	1.323(40)
r_e	2.14853(11)	2.14708(50)

Supplementary Table 2: Equilibrium Constants of the $J0_u^+$ State of $^{63}Cu_2$. All values are in cm^{-1} except r_e in Å. Values in parentheses denote one standard deviation in the last digit. T_e: equilibrium electronic term. ω_e: equilibrium vibrational constant. $\omega_e x_e$: equilibrium anharmonicity constant. B_e: equilibrium rotational constant. α_e: equilibrium vibration-rotation interaction constant. r_e: equilibrium internuclear separation.

	$^{63}Cu_2$				
Level	T_v	B_v	$D_v \times 10^9$	$q_v \times 10^6$	$H_v \times 10^{12}$
$I_1 1_u$	37410.7859(76)	0.112440(27)	1.59(22)	-1.456(47)	-0.913(51)
$I_2 1_u$	37700.1238(64)	0.113071(14)	0.413(53)	0.368(53)	

	$^{65}Cu^{63}Cu$			
Level	T_v	B_v	$D_v \times 10^9$	$q_v \times 10^6$
$I_1 1_u$	37406.6047(61)	0.110851(14)	4.854(69)	0.662(83)
$I_2 1_u$	37694.3950(64)	0.111445(18)	0.555(76)	0.156(93)

Supplementary Table 3: Apparent Molecular Constants of the $\mathbf{I1_u}$ State. All values in units of cm^{-1}. T_v: origin (sum of electronic term T_e and vibrational term $G(v)$). B_v: mean value of the rotational constant B in the vibrational state v. D_v: mean value of the quadratic centrifugal distortion constant D in the vibrational state v. q_v: mean lambda doubling constant q in the vibrational state v. H_v: mean value of the sextic centrifugal distortion constant H in the vibrational state v.

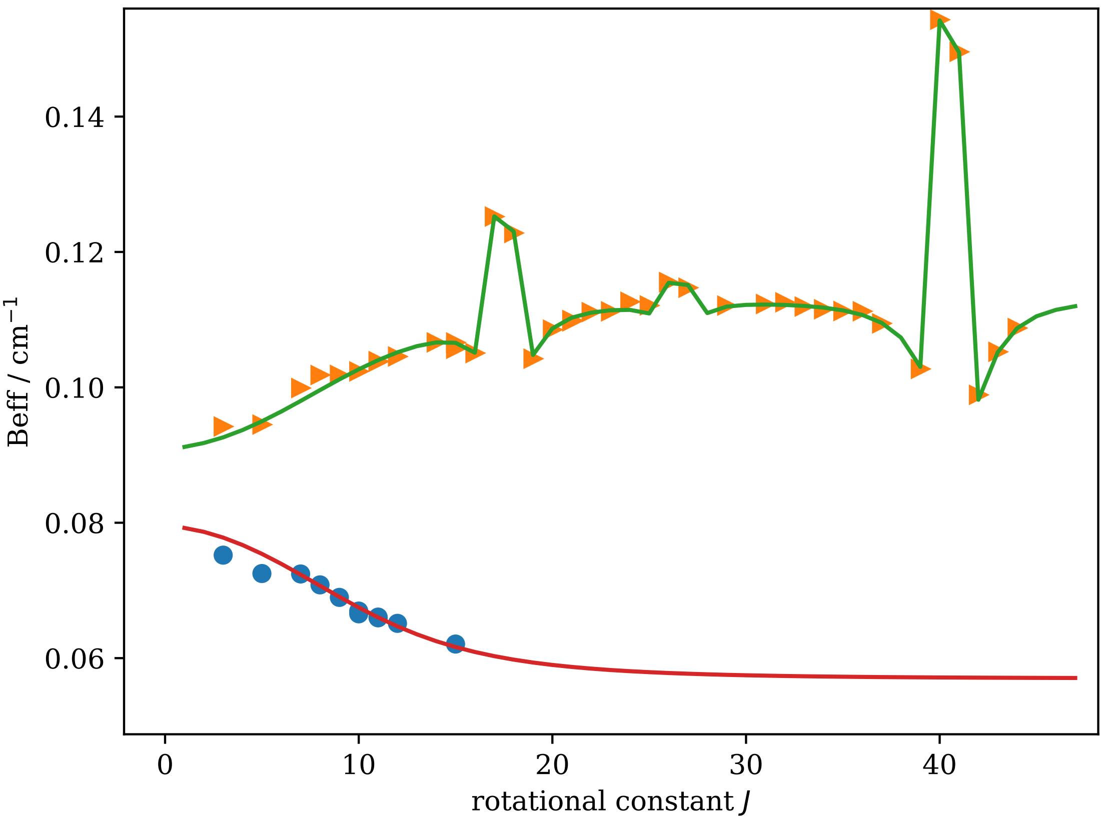

Supplementary Figure 1: Homogeneous perturbation of the J0_u^+ (v=2) ∼ $G_{67}0_u^+$ system. The effective rotational constants, $B_{\rm eff}$ vs. J are shown for J0_u^+ ($v = 2$) and $G_{67}0_u^+$ (upper and lower trace, respectively). The solid lines are obtained from the deperturbation analysis of the system. A crossing of the two levels at $J < 0$ is indicative for a homogeneous perturbation.[3,4] Perturbations of the J0_u^+ ($v = 2$) level with $N1, M1$ and $G_{68}0_u^+$ appear in addition at approximately $J = 18, 28$ and 40, respectively.

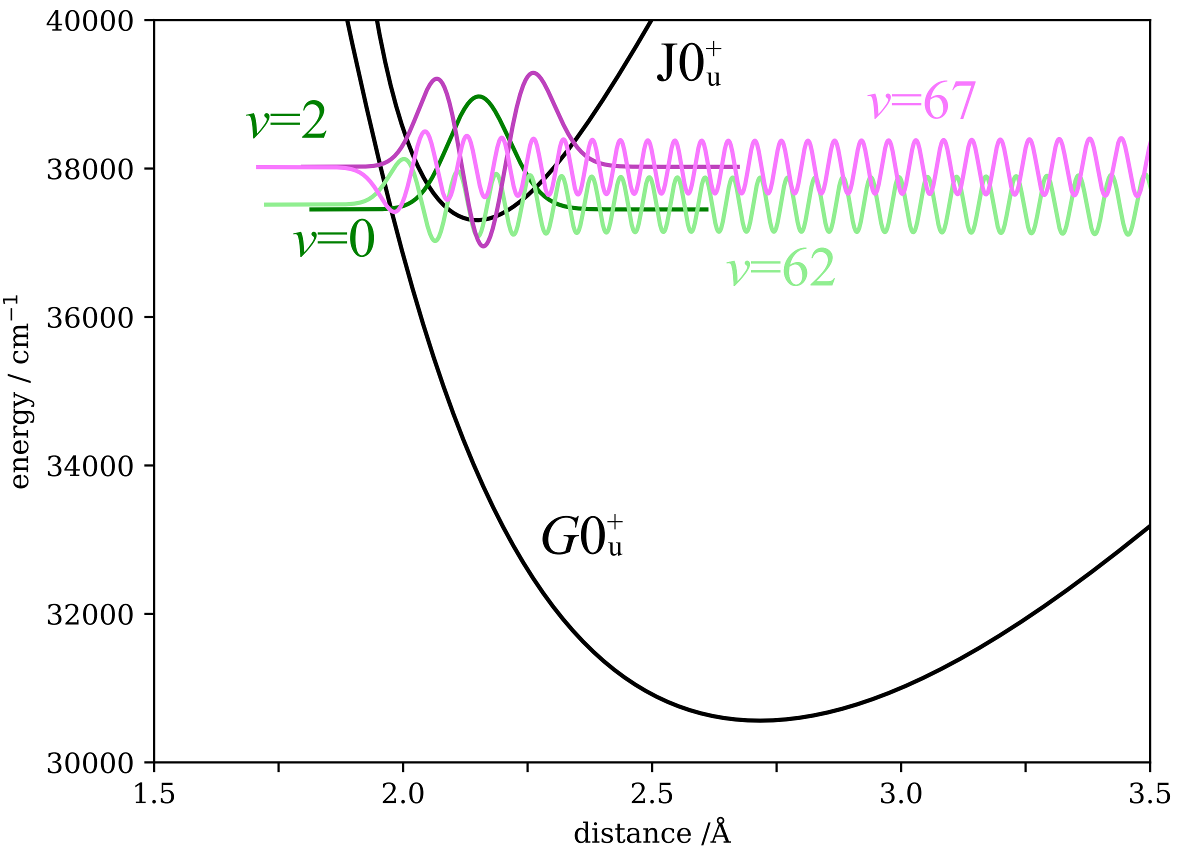

Supplementary Figure 2: Vibrational Overlap. The wavefunctions of the near resonant $G_{62}0_\mathrm{u}^+$ and $\mathrm{J}0_\mathrm{u}^+$ ($v = 0$) states shown in magenta and the $G_{67}0_\mathrm{u}^+$ and $\mathrm{J}0_\mathrm{u}^+$ ($v = 2$) shown in turqouise. The overlap integrals are very favorable in both cases. A ratio of 1:3:6 is computed for the overlap integrals between $G_{62}0_\mathrm{u}^+ \sim \mathrm{J}0_\mathrm{u}^+(v = 0) \approx G_{63}0_\mathrm{u}^+ \sim \mathrm{J}0_\mathrm{u}^+(v = 0)$: $G_{64}0_\mathrm{u}^+ \sim \mathrm{J}0_\mathrm{u}^+(v = 1) \approx G_{65}0_\mathrm{u}^+ \sim \mathrm{J}0_\mathrm{u}^+(v = 1) \approx G_{66}0_\mathrm{u}^+ \sim \mathrm{J}0_\mathrm{u}^+(v = 1)$: $G_{67}0_\mathrm{u}^+ \sim \mathrm{J}0_\mathrm{u}^+(v = 2) \approx G_{68}0_\mathrm{u}^+ \sim \mathrm{J}0_\mathrm{u}^+(v = 2)$ which is in good agreement with the values for the homogeneous perturbation strength between the $G_{xx}0_\mathrm{u}^+$ and $\mathrm{J}0_\mathrm{u}^+$ states deduced from the experiment shown in Supplementary Table 1.

SUPPLEMENTARY REFERENCES

1. Beck, M. *et al.* Rovibrational Characterization of High-Lying Electronic States of Cu_2 by Double-Resonant Nonlinear Spectroscopy. *J. Phys. Chem. A* **121**, 8448–8452 (2017).
2. Powers, D. E., Hansen, S. G., Geusic, M. E., Michalopoulos, D. L. & Smalley, R. E. Supersonic copper clusters. *The Journal of Chemical Physics* **78**, 2866–2881 (1983).
3. Bender, D. *UV-Laserspektroskopie gestörter Zustände des Bariumoxids* (University of Hannover, Thesis, Hannover, 1985).
4. Bender, D., Schaefer, S. & Tiemann, E. Laser spectroscopy of perturbed states of BaO in the region above 32 000 cm-1. *Journal of Molecular Spectroscopy* **116**, 286–314 (1986).
