# Supplementary material for: Spectroscopic disentanglement of the quantum states of highly excited Cu2
Source: Nat Commun. 2019 Jul 22;10:3270. doi: 10.1038/s41467-019-11156-2 (PMC6646321; doi:10.1038/s41467-019-11156-2)
Supplement: Supplementary file 3 — Description of Additional Supplementary Files [file 41467_2019_11156_MOESM3_ESM.docx]

**Description of Supplementary Files**

**File Name:** **Supplementary Data 1**

**Description:** Observed transitions and assignments used to determine molecular constants in Supplementary Table 1. Transitions and residuals are in cm-1. Original raw data files are provided upon request from the corresponding author.
